# Microglial morphometric analysis: so many options, so little consistency

**DOI:** 10.3389/fninf.2023.1211188

**Published:** 2023-08-10

**Authors:** Jack Reddaway, Peter Eulalio Richardson, Ryan J. Bevan, Jessica Stoneman, Marco Palombo

**Affiliations:** ^1^Division of Neuroscience, School of Biosciences, Cardiff University, Cardiff, United Kingdom; ^2^Hodge Centre for Neuropsychiatric Immunology, Neuroscience and Mental Health Innovation Institute (NMHII), Cardiff University, Cardiff, United Kingdom; ^3^UK Dementia Research Institute, Cardiff University, Cardiff, United Kingdom; ^4^Cardiff University Brain Research Imaging Centre (CUBRIC), School of Psychology, Cardiff University, Cardiff, United Kingdom; ^5^School of Computer Science and Informatics, Cardiff University, Cardiff, United Kingdom

**Keywords:** microglia, microglia morphology, cellular morphological changes, machine learning, hierarchical cluster analysis, neuroimmune methods

## Abstract

Quantification of microglial activation through morphometric analysis has long been a staple of the neuroimmunologist’s toolkit. Microglial morphological phenomics can be conducted through either manual classification or constructing a digital skeleton and extracting morphometric data from it. Multiple open-access and paid software packages are available to generate these skeletons via semi-automated and/or fully automated methods with varying degrees of accuracy. Despite advancements in methods to generate morphometrics (quantitative measures of cellular morphology), there has been limited development of tools to analyze the datasets they generate, in particular those containing parameters from tens of thousands of cells analyzed by fully automated pipelines. In this review, we compare and critique the approaches using cluster analysis and machine learning driven predictive algorithms that have been developed to tackle these large datasets, and propose improvements for these methods. In particular, we highlight the need for a commitment to *open science* from groups developing these classifiers. Furthermore, we call attention to a need for communication between those with a strong software engineering/computer science background and neuroimmunologists to produce effective analytical tools with simplified operability if we are to see their wide-spread adoption by the glia biology community.

## Introduction to microglia and the functional relevance of their morphology

The central nervous system (CNS) is populated with tissue specific macrophages, termed microglia, that comprise 10–15% of cells in the adult brain (Li and Barres, 2018; Morimoto and Nakajima, 2019). In rodent studies (at E8 timepoint), primitive macrophages from a pool in the yolk sac colonize the embryonic brain, whilst in humans their presence appears from 4.5 weeks into gestation (Monier et al., 2007; Thion et al., 2018). Upon maturation of the blood brain barrier (BBB), microglia are confined to the CNS (providing BBB integrity is not compromised) and self-renew throughout adulthood (Daneman and Prat, 2015; Lenz and Nelson, 2018). Microglia are susceptible to a wide range of factors that can influence their function. Since their turnover is slow, adverse deviations from the homeostatic environment such as those observed in brain injury, neurological disease and stress, can have long lasting effects on their functional capacity in the brain (Ziebell et al., 2017b).

Historically, microglia in the healthy (non-diseased/injured) adult brain have been termed “resting” which is a misnomer. Microglia in a healthy adult brain participate in CNS homeostasis and survey their local environment for pathogens, apoptotic/necrotic cells, neurofibrillary tangles, amyloid plaques, and deoxyribonucleic acid (DNA) fragments (Nimmerjahn et al., 2005; Bolmont et al., 2008; Ohm et al., 2021). Furthermore, by releasing diffusible factors (e.g., cytokines and trophic factors) and conducting phagocytosis/trogocytosis, microglia directly support healthy brain function and CNS development maintaining homeostasis, support of neurotransmission, remodeling the extracellular matrix, neuronal maintenance, the regulation of neurogenesis and facilitation of synaptic pruning, long term potentiation (LTP) and long-term depression (LTD) (Paolicelli and Gross, 2011; Frost and Schafer, 2016; Salter and Stevens, 2017; Madry et al., 2018b; Weinhard et al., 2018a; Crapser et al., 2020; Nguyen et al., 2020; Venturino et al., 2021; Buchanan et al., 2022). Homeostatic microglia react via their arrays of trans-membrane receptors to damage or pathogen associated molecular pattern (DAMPs or PAMPs) (Kyrargyri et al., 2020). Upon encountering immunogenic stimuli, microglia synthesize and release cytokines and migrate following chemotactic gradients to sites of injury and/or damage where they modulate secondary injury and facilitate repair (Thameem Dheen et al., 2007; Bachiller et al., 2018).

**FIGURE 1:**
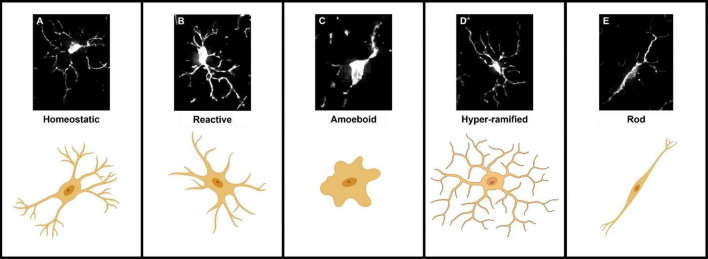
Representations of the five microglial morphologies. **(A)** Homeostatic microglia characterized by their long complex processes and small somas. **(B)** Reactive microglia processes have retracted and decreased in number, somas also appear larger and rounder. **(C)** Amoeboid microglia lack any processes and are morphologically indistinguishable from blood-borne macrophages. **(D)** Hyper-ramified “bushy” microglia an intermediate morphology between homeostatic and reactive, in which microglia have retracted and begun to thicken their processes. **(E)** Rod-shaped microglia adopt a highly polarized morphology characterized by their long thin somas and long extended processes.

Microglia are highly dynamic cells that display multivariate ontogeny, morphology, motility, transcriptomes, and metabolic profiles. The many layers of complexity, intrinsic and extrinsic determinants and the spatiotemporal context confer microglial functional roles. Despite this complexity morphological states of microglia have often been used as a proxy measure of their functional state. After over a century of study, the morphological phenotypes of microglia, although diverse, can be loosely grouped based on a variety of features into: homeostatic, hyper-ramified, reactive, amoeboid and rod (Sierra et al., 2019). Most microglia in the healthy adult brain display homeostatic morphologies with numerous ramifications, small somas, long processes and highly arborised branches surveying a relatively large area in the search for signs of infections or neuronal distress (cell area: 200–8,000 μm^2^, skeleton length: 200–350 μm and cell solidity: 0.25–0.3) (Figure 1A; Glenn et al., 1992; Leyh et al., 2021). Upon detection of a stimulus, the cytoskeleton rearranges driving microglia to adopt a reactive morphology. During this transition microglia adopt an intermediate morphology termed hyper-ramified or “bushy” in which they exhibit increased process length, volume and complexity (Figure 1D; Ziebell et al., 2015; Fernández-Arjona et al., 2017). Reactive microglia are characterized by larger somas and thicker less branched processes compared to the ramified morphotype (cell area: 200–400 μm^2^, skeleton length: 100–300 μm and cell solidity: 0.3–0.35) (Figure 1B; Tynan et al., 2010; Ziebell et al., 2015; Madry et al., 2018a; Savage et al., 2019; Leyh et al., 2021). The reactive morphotype is associated with microglia that are no longer solely surveying for signaling molecules associated with damaged neurons or pathogens, but also the production of inflammatory cytokines, phagocytosing of cellular debris and/or migrating to sites of injury (Li et al., 2007; Lynch, 2009; Lannes et al., 2017). In scenarios such as neuroinflammation, where immunogenic molecules are in high concentrations for a sustained period of time, reactive microglia retract their processes entirely and adopt a round amoeboid morphotype that is indistinguishable (at least morphologically, there are some protein markers such as Hexb which are microglia specific) from infiltrating peripheral macrophages (cell area: 50–150 μm^2^, skeleton length: 25–50 μm and cell solidity: 0.4–0.5) (Figure 1C; Giulian and Baker, 1986; Parakalan et al., 2012; Jurga et al., 2020; Masuda et al., 2020; Leyh et al., 2021). Microglia can also adopt another morphology; where their processes are highly polarized with little arborisation and their somas skinny and elongated, meaning the cells resemble rods (cell area: 300–700 μm^2^, skeleton length: 200–350 μm and cell solidity: 0.25–0.3) (Figure 1E; Leyh et al., 2021). Rod microglia align in parallel with damaged neurons post-injury and appear to facilitate either their repair or further breakdown (Taylor et al., 2014; Holloway et al., 2019; Giordano et al., 2021). Historically, the nomenclature used to describe microglial morphology has been extremely varied with some terms being used interchangeably and with some researchers preferring one term over another. Recently, in an effort to achieve a degree of concordance in microglial morphological nomenclature, researchers in the field came together to standardize the terminology used to describe microglial functional states (Paolicelli et al., 2022). In the course of this review where appropriate we have adhered to this new nomenclature. However, when presenting the work and models developed by other researchers, we have retained their original nomenclature. To avoid confusion Table 1 provides a summary of terms used to describe microglial morphotypes during this review.

**TABLE 1:** The varied names/nomenclature found within the literature used to describe microglial morphotypes and how they relate to the terms defined by Paolicelli et al. (2022).

Term defined by Paolicelli et al. (2022)	Alternative names
Homeostatic	Resting
	Ramified
	Non-active
	Surveillant
	M0
Hyper-ramified	Bushy
Reactive	Activated
	Inflammatory
	M1/M2
	Hypertrophied
	Deramified
	Disease associated microglia (DAM)
Amoeboid	Fully active
	Phagocytic

Given the well-established literature on microglial morphology and its relation to function; researchers often use assessments of microglial morphology as a proxy for assessing the functional state of microglia in the brain during development, during disease progression, post-injury, accompanying genetic alterations, or following behavioral paradigms. Microglial morphological phenomics can be combined with additional measures of microglial activity such as assaying PSD95 (a post-synaptic scaffolding protein) uptake to quantify microglial synaptic phagocytosis and measuring increases in the expression of pro-inflammatory markers such as: CD45, CD68 and MHCII (Tynan et al., 2010; Morrison et al., 2017; Hopperton et al., 2018; Cengiz et al., 2019; Paasila et al., 2019; Sellgren et al., 2019; Sinha et al., 2021).

## Methods for labeling microglia

Morphological assessment of microglia requires a pan-microglial marker which covers the entire cell (including all processes and soma). Immunohistochemistry targeting the cytoskeleton associated monocyte-specific marker ionized calcium binding adaptor molecule 1 (IBA1) has long been considered the gold standard for labeling microglia for morphometric analysis (Ito et al., 1998; Ahmed et al., 2007). Other pan-microglial markers include CD11b, CX3CR1, and CD68 all of which, like IBA1, are expressed by infiltrating perivascular and meningeal macrophages (Korzhevskii and Kirik, 2016). The non-specific labeling of said markers does not always represent an issue for morphological assessment as perivascular, meningeal and border-associated macrophages are readily identifiable based upon their localisation and peripheral macrophages are not present in the healthy CNS thanks to the BBB (Zhao et al., 2015; Utz and Greter, 2019; Yang et al., 2019). However, in models of disease, injury and infection such as Alzheimer’s disease (AD), traumatic brain injury (TBI), ischemia and streptococcal meningoencephalitis where the BBB is compromised/leaky, peripheral macrophages can enter the brain and thus can be misidentified as microglia during morphological assessment (Polfliet et al., 2001; Chodobski et al., 2011; Sweeney et al., 2018; Gres et al., 2019; Nian et al., 2020). Recently TMEM119 (Satoh et al., 2016), P2RY12 (Butovsky et al., 2014) and SiglecH (Konishi et al., 2017) have been identified as a microglial markers which are not expressed by macrophages, however, they have not usurped IBA1 as the microglial marker of choice for morphometrics in part due to limited antibody availability and reports of down regulation post-microglial activation (Bennett et al., 2016; Satoh et al., 2016). Reporter knock-in animals where a microglial marker is co-expressed/tagged with a fluorescent reporter such as GFP or tdTomato can be used instead of immunohistochemistry for labeling microglia for morphological assessment. Both myeloid (CX3CR1^GFP^) and microglial (TMEM119^GFP^, Sall1^GFP^, IBA1-EGFP, and Hexb^TdTomato^) specific reporter mice have been developed (Garcia et al., 2013; Koso et al., 2016; Kaiser and Feng, 2019; Masuda et al., 2020; VanRyzin et al., 2021). However, care must be taken to select a suitable marker whose expression is relatively consistent between experimental groups (i.e., naïve vs. injured). TMEM119 and Sall1 expression have both been shown to decrease during prolonged periods of microglial activation IBA1, CD11b, CD68, and CX3CR1 are increased under the same conditions (Roy et al., 2006; Zhang et al., 2018; Jurga et al., 2020; Shi et al., 2021). Hexb appears to be more stable under similar conditions (Bennett et al., 2016; Masuda et al., 2020). Additionally, following its first description concern has been raised over TMEM119’s microglial specificity, for example in the retina TMEM119 was found to be expressed by both microglia and Müller cells (Su et al., 2019). Moreover, during early development (4.5 weeks), TMEM119 does not label microglia and instead may be specific to osteoblasts (Bartalska et al., 2022). Furthermore, some models such as the CX3CR1 gene knock-in, must consider the impact of partial deficiencies in their expression that the model induces, for example in APP-PS1 mice partial CX3CR1 deficiency reduces plaque deposition (Hickman et al., 2019), and total CX3CR1 deficient microglia display premature aging phenotypes (Gyoneva et al., 2019; Table 2).

**TABLE 2:** Summary of methods to label microglia *in vivo* with their respective advantages and disadvantages.

Category	Marker	Function	Advantages	Disadvantages
Myeloid	CD11b	Subunit of complement receptor 3 Chen et al., 2008	Well established markers	Expressed by peripheral myeloid cells and infiltrating macrophages
	CD68	Peptide transport/antigen processing Chistiakov et al., 2017		
	CX3CR1	Chemokine receptor Lee et al., 2018	Plethora of widely available antibodies	
	IBA1	Cytoskeleton calcium-binding adaptor Ohsawa et al., 2004		
Microglial	HexB	Lysosomal enzyme subunit Masuda et al., 2020	Stable expression post-microglial activation Expressed only by microglia	Limited commercially available antibodies
	P2ry12	Adenosine diphosphate receptor Gómez Morillas et al., 2021	Expressed only by microglia	Limited commercially available antibodies Decreased expression with sustained microglial activation
	SiglecH	Phagocytosis receptor Kopatz et al., 2013		
	TMEM119	Yet to be established Bennett et al., 2016	Expressed only by microglia (in the adult brain)	Limited commercially available antibodies Decreased expression with sustained microglial activation Repressed during development Expressed by retinal Müller cells
Reporter mice	CX3CR1^GFP^	Chemokine receptor Lee et al., 2018	Well established marker	Expressed by peripheral myeloid cells and infiltrating macrophages Impaired signaling of receptor compared to WTs
	HexB^TdTomato^	Lysosomal enzyme subunit Masuda et al., 2020	Expressed only by microglia High specificity Stable expression post-microglial activation	
	Sall1^GFP^	Zinc finger transcriptional repressor Buttgereit et al., 2016	Expressed only by microglia (in the adult brain)	Decreased expression with sustained microglial activation
	TMEM119^GFP^	Yet to be established Bennett et al., 2016		Decreased expression with sustained microglial activation Repressed during development Expressed by retinal Müller cells

## Tools for imaging stained/labeled microglia *in situ*

Confocal laser scanning microscopy is the favored approach to image fluorescently labeled microglia in tissue sections. Care must be taken to select an appropriate section thickness which ensures the full 3D architecture of microglia is captured but is thin enough to facilitate adequate antibody penetration if immunohistochemistry is being used to label cells. Typically, with appropriate retrieval and fixation strategies, section thickness of up to 150 μm can be used which will sufficiently capture typical microglial territorial depth (Davis et al., 2017; Leyh et al., 2021). Furthermore, to ensure only fully intact microglia are imaged care should be taken to avoid microglia on the tissue border which are likely incomplete. Whilst traditional confocal microscopes are more than capable of imaging small field images of microglia in tissue sections, the advent of automated slide scanners such as the ZEISS Axioscan 7 and Olympus VS120 have revolutionized the acquisition of microglial images for morphometric analysis. These microscopes enable entire brain regions or whole sections to be imaged resulting in thousands of microglial images being acquired per animal with reduced sampling bias. Large microglial image datasets can also be obtained via light-sheet microscopy used in conjunction with methods to clear brains containing fluorescently labeled microglia. This approach ensures intact microglial architecture and enables global analysis of microglial morphotypes without sectioning artifacts; however, with the caveat that the shrinkage which occurs during sample preparation will alter microglial profiles (Bekkouche et al., 2020; Vulders et al., 2021). Furthermore, light-sheet technology has modest spatial resolution which does pose an issue for researchers wanting to capture finer microglial processes in their morphometric analysis (Watkins and St. Croix, 2018; Chen et al., 2022).

## Methods for assessing morphology

Parallel improvements in high performance cluster computing, machine learning algorithms and image classification frameworks built on deep learning architectures show that the field of imaging in biomedical sciences is entering a new age of high throughput analysis (Barragán-Montero et al., 2021; Castiglioni et al., 2021; Zerouaoui and Idri, 2021). However, these developments bring with them new challenges in how to assess microglia morphology in a manner that capitalizes upon the large microglial image datasets being acquired. We begin by describing the different methods for microglial classification and morphological analysis, starting with the low-throughput methods and ending with classification pipelines capable of analyzing thousands of cells.

### Manual classification

**FIGURE 2:**
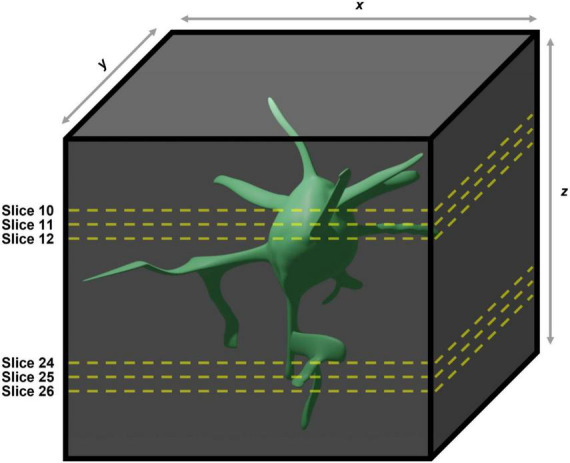
Demonstration of the caveats associated with using two-dimensional maximum intensity projections generated from three dimensional images for manual quantification of microglial morphologies. In the case of the example given the large soma contained within slices 10–12 in the maximum intensity projection will obscure the large process contained within slices 24–26, meaning they will not be included in any analyses performed.

Manual classification of microglial morphology using maximum intensity projection images generated from 3D images of microglia is a simple, commonly used method to assess microglial activation status in a given context. In manual classification, a trained “scorer” is given an image dataset typically containing 50–250 microglia per subject (Table 3) they then class each cell as being either homeostatic, reactive, amoeboid or rod-shaped whilst being blinded to the experimental group/animal they are from. Classification is done according to a set of criteria for each morphology: homeostatic–large surveying area with thin highly arborised processes; reactive–enlarged soma with thicker and less branched processes; ameboid–no processes with large soma; rod–long polarized processes with a thin soma. Whilst this approach requires no specialist software and can be a relatively rapid method for analysis once the initial “scorer” training has been completed, the criteria are very subjective and effective blinding is not always possible when studying disease and injury models with distinctive histopathological features. Subjectivity becomes an even greater issue if researchers wish to include in their analysis the hyper-ramified morphotype (Figure 1D), which is difficult to identify/separate from homeostatic and reactive morphotypes due to it being a transitory stage between the two (Morrison and Filosa, 2013; Holloway et al., 2019). Another significant challenge regarding manual morphological assessment is loss of data through dimensionality reduction. Here, trying to view a 3D object as a 2D object is done by viewing z-stacked images as a maximum intensity projection. This is quick and computationally simple, for example, ImageJ^1^ contains the 3D Project function which does this. Nonetheless, the resulting image hides processes that are obstructed by the cell body or other thicker processes. Figure 2 shows a schematic of this issue. Whilst the classification could, in theory, be done by a scorer scrolling through the z-plane, this proves to be challenging and impractical.

**TABLE 3:** A non-exhaustive sampling of the number of cells assessed in recent highly cited papers using using the four most common methods for microglial morphological analysis: manual classification, fractal analysis, manual/semi-automated reconstruction, and automated reconstruction.

References	Area of research	# Cells assessed
**Manual classification**		
Diz-Chaves et al., 2012	Psychological stress	28 counting frames per animal
Ziebell et al., 2017a	Traumatic brain injury	100 cells (16 counting frames) per animal
Davies et al., 2017	Alzheimer’s disease	∼200 cells per individual
DeWalt et al., 2018	Blast injury	∼50 cells per ROI
Martini et al., 2020	Alzheimer’s disease	5 counting fames per section
Vega-Rivera et al., 2021	Psychological stress	50 cells per animal
Bachstetter et al., 2015	Alzheimer’s disease	5 counting fames per section
Sardari et al., 2020	Ischemia	6 counting frames per section
Hoeijmakers et al., 2017	Alzheimer’s disease	4 counting frames per section
Villapol et al., 2017	Traumatic brain injury	2–5 counting frames per animal/ROI
**Fractal analysis**		
Sołtys et al., 2001	Lesion injury	145 cells in total
Morrison et al., 2017	Traumatic brain injury	24 cells per animal/ROI
Daly et al., 2022	Nutrition	20 cells per animal
Kuo et al., 2021	Ischemia	30 cells per animal
Francistiová et al., 2022	Microglial biology	8 cells per condition
Bido et al., 2021	Parkinson’s disease	30 cells per animal
Young et al., 2021	Ischemia	3 cells per animal/ROI
Fletcher et al., 2020	Parkinson’s disease	27 cells per animal
Green et al., 2022a	Microglial biology	478 cells in total
**Manual/Semi-automated reconstruction**		
Kongsui et al., 2014	Microglial biology	5 cells per ROI
Bolton et al., 2017	Neurodevelopment	45 cells in total
Ali et al., 2019	Environmental enrichment	10–20 cells per condition/ ROI
Franco-Bocanegra et al., 2021	Alzheimer’s disease	15 cells per individual
Franciosi et al., 2012	Huntington’s disease	∼1000 cells per condition
Cengiz et al., 2019	Parkinson’s disease	5–10 cells per animal
Gildawie et al., 2020	Neurodevelopment	200-600 cells per animal
Gober et al., 2022	Schizophrenia	30 cells per individual
Chaaya et al., 2019	Behavioral neuroscience	9 cells per ROI
Weinhard et al., 2018b	Neurodevelopment	60 cells per condition
Fujikawa and Jinno, 2022	Psychological stress	255 cells in total
**Automated reconstruction**		
Verdonk et al., 2016	Microglial biology	20,000 cells per condition
Kozlowski and Weimer, 2012	Microglial biology	10,000 cells per condition
Pelgrim et al., 2022	Chronic obstructive pulmonary disease	12 cells per ROI
Elzinga et al., 2022	Nutrition/Microglial biology	∼5,000 cells per condition
Martinez et al., 2023	Microglial biology	∼5,000 cells in total
Leyh et al., 2021	Ischemia	15,786 cells in total

ROI (region of interest). Demonstrating the within method variability of sample number and the advantages afforded by automated reconstruction methods.

A major drawback of manual classification is the introduction of inter-rater biases. Morphology is complex; important parameters including branch angle, branch thickness and soma size, are challenging for a human investigator to accurately gauge. The boundaries between one morphology and the next can be very subtle (for example between homeostatic and hyper-ramified). Furthermore, there exists no standard guide for microglia classification, meaning that one person’s homeostatic microglia is another person’s reactive microglia. This lack of consistency can lead to an extreme disparity in classification between observers. For example in a recent study, Chaaya et al. (2019) classed a population of cells with large processes and arborisation as ameboid whereas by many other researchers’ rubrics these cells would be classified as having a reactive morphology. This discrepancy serves the point that manual classification struggles with inter-rater reproducibility, especially between different research groups. We must also consider the substantial time commitment that manual classification requires, even when sampling a small population of 100 microglia per animal, analysis will take days and require the undivided attention of the scorer. In response to these issues and the desire to assess microglial morphology in a less subjective manner, methods have been developed to extract morphometrics from microglia.

### Fractal analysis

**FIGURE 3:**
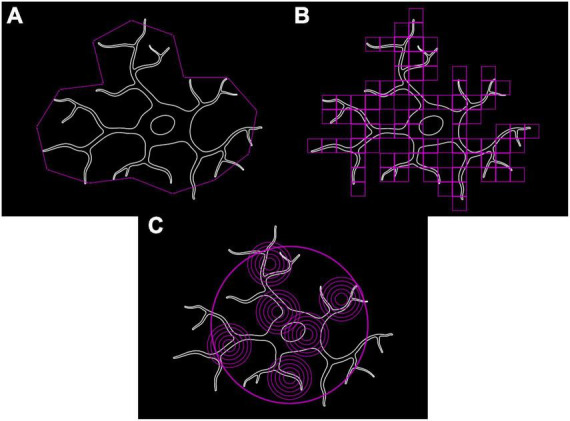
Methods for calculating fractal dimension used to quantify microglial complexity. **(A)** Classical length/caliper method. **(B)** Box counting method. **(C)** Mass-radius method.

Microglia, with their branching trees and formation of complex patterns, are fractals as per the definition of Benoît (1975) and therefore can have their complexity and structural variation quantified using fractal methods (Mandelbrot, 1967). A fractal’s complexity can be quantitatively assessed through calculating its fractal dimension (*D*), using either a length or a mass method. The classical length or caliper method measures the perimeter of an object with different lengths of ruler (e.g., 5, 10, and 20 pixels). Log(perimeter) is plotted against log(ruler length) to which a straight line is fitted whose slope (*S*) is used to calculate *D* (where *D* = 1–*S*) (Figure 3A; Jelinek et al., 2005). Box Counting is another length-based method for obtaining *D*, where a series of increasingly fine grids are applied to the image and the number of grid boxes covering the image counted (Figure 3B). D is calculated as follows:


$$D=\frac{l\,o\,g\,\left(N\right)}{l\,o\,g\,\left(1/G\right)}$$

Where *N* is the number of squares that cover the pattern and *G* is the grid size (Smith et al., 1996; Rajković et al., 2017). Mass methods utilize boxes or circles of varying sizes placed at random points along the shape’s perimeter and the number of border pixels contained within the shape are counted (Figure 3C). The slope (*S*) is calculated as follows:


$$S=\frac{l\,o\,g\,\left(p\right)}{l\,o\,g\,\left(w\right)}$$

Where *p* is the number of pixels in the applied box/circle and *w* is the applied box width or circle diameter (Smith et al., 1996; Jelinek and Fernandez, 1998). This slope is then used to calculate *D*.

**FIGURE 4:**
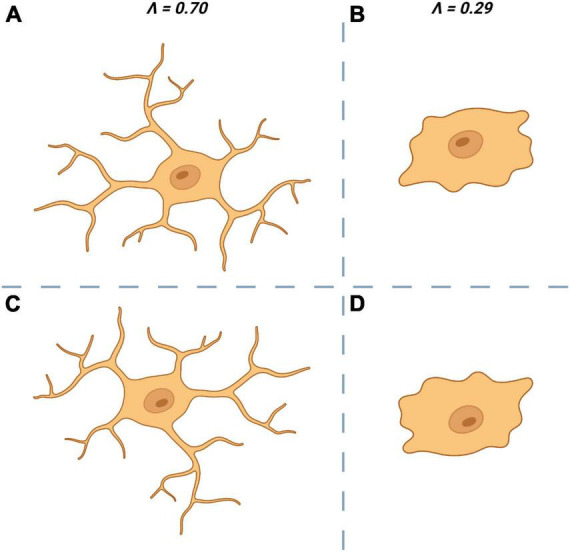
Lacunarity (∧ or *L*) a measure of rotational invariance is used to quantify the non-uniformity of microglia. The top left cell **(A)** is heterogenous and is rotationally invariant. When rotated by 180° **(C)** it looks dissimilar to the original **(A)** which is reflected by having a ∧ close to 1. The top right cell **(B)** is more homogenous and is less affected by rotation **(D)** which results in a ∧ closer to 0.

Structural variation or lacunarity (*L*) is another metric obtainable through fractal analysis and is a quantifiable measure of rotational/translational invariance (non-uniformity) (Figure 4). As with *D* there is little consensus on the “best” approach to calculate a fractal’s *L*. One common approach is to apply a series of increasingly fine grids to the image and then measuring the average number of pixels per square for each size of grid. Normalization (standard deviation/mean^2^) of these average pixel counts give the coefficient of variation, the average of which across all grid sizes is *L* (Smith et al., 1996; Jelinek and Fernandez, 1998).

The first implementation of fractal analysis to study microglial morphology (Sołtys et al., 2001), expanded existing methods used for morphological assessment of neurons, astrocytes and oligodendrocytes (Montague and Friedlander, 1989; Huxlin et al., 1992; Smith and Behar, 1994). In its simplest form, fractal analysis is performed on a one-pixel outline of a 2D microglial image generated through manual thresholding in ImageJ and plugins such as FracLac. A new fractal analysis plugin, MULTIFRAC^2^; released in Torre et al. (2020), still requires manually generated cell outlines, but can perform both 2D and 3D dimensional fractal analysis with researchers typically assessing 5–150 cells per subject (Table 3). Regardless of the method or software employed, all produce estimates of *D* and *L*. Unlike raw morphometrics, the parameters generated by fractal analysis do not lend themselves to simple interpretation as to the morphotype of the cell they are obtained from. Sołtys et al. (2001) along with Jelinek et al. (2008) used manual classification alongside fractal analysis to determine that both *D* and *L* decrease as a microglia trend toward activation. The one-pixel outlines generated during fractal analysis can be used to measure a limited range of raw morphological parameters such as convexity (convex hull perimeter/cell perimeter), solidity (cell area/convex area) and form factor (4π*area/perimeter^2^), which are often presented alongside *D* and *L* values in studies using fractal analysis to quantify microglial morphological changes (Sołtys et al., 2001; Fernández-Arjona et al., 2017; Morrison et al., 2017). When fractal analysis was first implemented to analyze microglial morphometrics by Sołtys et al. (2001) it was truly cutting edge and was one of the only methods available to extract quantitative measures of cellular morphology. However, in the intervening 20 years the emergence of software packages (detailed in the next section), which facilitate both manual and automatic microglial tracing, have supplanted fractal analysis as the “gold standard” for quantitively assessing cellular morphology. These newer methods can produce detailed multi-dimensional datasets to quantify microglial morphology, opposed to the one or two parameters generated by fractal analysis. Furthermore, fractal analysis suffers from issues of reproducibility and user bias due to the need for manual thresholding of images and operator driven selection of cells. Despite these issues, fractal analysis could feasibly be incorporated into the semi-/fully automated morphological analysis platforms discussed later in this review, especially as some of these software packages do not require the operator to select cells for analysis. This inclusion would increase the dimensionality of the datasets they generate through the inclusion of *D* and *L*, which could be particularly useful for machine learning driven classification and/or cluster analysis.

### Manual approaches

Several manual tracing software solutions are available to researchers ranging from free open-source ImageJ plugins such as Simple Neurite Tracer [SNT, (Arshadi et al., 2021)] or Analyze Skeleton (Arganda-Carreras et al., 2010) to commercial packages Neurolucida 360^®^ (MBF Bioscience) and Imaris Microscopy Image Analysis software (Oxford Instruments) which are feature rich but very expensive (>$15,000 for typical academic license). All these software packages regardless of cost or number of features enable the operator to manually trace a projection generated from a 3D microglial image. Of course, this comes with the same caveats surrounding 2D projections discussed previously but has the capacity that the original 3D image can be referred to, enabling the user to solve any ambiguity such as overlapping processes. In some cases, particularly with the more advanced and feature heavy platforms, semi-automated is a more apt description as the manual tracing is often guided by the software or an imprecise trace is generated by the software which the user can then manually tweak and edit themselves. The Imaris Microscopy Image Analysis software package generates these automatic editable traces by allowing the user to set “starting points” and “seed points,” along which the microglial structure is detected. Whilst its developers regard this feature as “automatic” we believe semi-automatic is more appropriate. From experience manual edits of “start” and “seed” points is almost always required and calls for extensive user input when compared to the fully automated approaches that we will go onto discuss later. Nonetheless, packages like Imaris Microscopy Image Analysis and Neurolucida 360^®^ provide highly precise and detailed traces (albeit requiring some manual editing) of microglia from which morphometrics can be extracted. All methods of manual tracing include a level of bias; from personal experience and speaking with peers, researchers performing manual tracing tend to select cells to trace that are “easy” e.g., are isolated with little overlap with other cell’s processes. This bias, whilst understandable, can severely impact the morphometric datasets generated, particularly when studying diseases such as AD and TBI where clusters of overlapping and intercalated microglia are expected around plaques and injury sites, respectively.

### Fully automated approaches

Several approaches for truly automated tracing have emerged recently and are beginning to challenge the dominance of manual and semi-automated methods to sample microglial morphometrics. Examples of automated tracing platforms currently available to researchers include the MATLAB based 3DMorph,^3^ (York et al., 2018) GliaTrace,^4^ (Abdolhoseini et al., 2019), MIC-MAC (Salamanca et al., 2019), and Microglia Morphology Quantification Tool,^5^ (Heindl et al., 2018) and standalone platforms such as Vaa3D,^6^ (Peng et al., 2010) and Acapella [PerkinElmer Technologies, USA, (Verdonk et al., 2016)]. Despite their differences, all these approaches to automated tracing revolve around two key processes: segmentation and skeletonisation. Segmentation separates cells out from its neighbors and addresses the major issue of cells and their processes overlapping in three-dimensional space (Al-Kofahi et al., 2018). Many automated quantitative methods of assessing microglial morphology require cells in isolation and are therefore dependent upon adequate segmentation. If cells cannot be segmented correctly then much of the further downstream analysis will either fail or generate inaccurate representations of the cells. As with many other aspects of microglia analysis, early attempts at segmentation used software developed for use with other cell types, such as Fogbank (breast epithelial cells), FastER (HeLa cells, blood progenitor cells and embryonic stem cells) and Cell-Profiler (*Drosophila* Kc167 cells and human HT29 cells) (Vicar et al., 2019). However, all these programs were originally developed for rounder cells with more homogenous profiles, and thus struggle to adequately segment highly arborised microglia whose processes extensively overlap in three-dimensions. Several microglia-specific segmentation algorithms have now begun to emerge in recent years; the vast majority utilize a manually set threshold which generates a binary image separating the cells from background. In the case of 3DMorph, segmentation begins with pre-processing in which the image is denoised and filtered to remove staining artifacts. This is followed by the identification of areas of highest staining intensity termed local minima, which are used as cell seed points and generally correspond to the center of the soma. From the seed point the intensity of the pixels in the surrounding area is compared to a user defined threshold. Once the intensity of the selected voxels hits a set threshold level the cell outline is drawn, and it is digitally separated from the background and neighboring cells from which morphometrics extracted. Post-segmentation the isolated cell’s internal skeleton can be constructed through a skeletonisation algorithm from which additional morphometrics can be extracted covering the cell’s ramification and branching hierarchy. The process of skeletonisation can broadly be divided into two main methods: the first, the method implemented by the scikit-image algorithm (van der Walt et al., 2014),^7^ is termed morphological thinning, and works by looping iteratively to delete border pixels with the condition that pixel removal does not break the connectivity of the shape (Guo and Hall, 1989; Lam et al., 1992). The second skeletonisation method is to generate a distance transform of the image or a medial axis transformation, during which pixels are labeled with their distance from the nearest boundary and the local maxima, representing the skeleton (Blum, 1967). As with segmentation, producing skeletons for cells with complex morphologies is a task which has been optimized and developed for use in the analysis of cell types other than microglia. Existing programs such as ImageJ’s Analyze Skeleton (Arganda-Carreras et al., 2010) and Python’s scikit-image have been co-opted for use in microglial analysis.

One of the primary advantages of automated tracing is that it requires minimal user input, following image acquisition (which itself can be automated using slide scanners), images can be processed via macros and batch processing before being fed into the automatic tracing software. With time no longer being an issue for the operator, this enables larger populations of microglia to be traced and the subsequent generation of larger microglial morphological datasets (>10,000 cells, Table 3). A review of the current microglial tracing literature found that researchers using manual/semi-automated tracing methods tend to trace between 5 and 500 microglia per animal (Table 3). These relatively low numbers are sufficient to detect morphological changes models of CNS injury and disease such as TBI (Donat et al., 2017; Ziebell et al., 2017a), AD (Hansen et al., 2018; Leng and Edison, 2021), and ischemia (Lai and Todd, 2006; Heindl et al., 2018; Zhang, 2019) where mass microglial activation is observed in biological scenarios. However, where more subtle changes in activation are expected such as following behavioral stressors, larger datasets are essential so that the reactive population of microglia are not “missed.” Furthermore, the use of automated tracing greatly reduces the user bias associated with manual tracing. There is no influence of the researcher upon which microglia are traced meaning there is no preference for easy to trace microglia as described previously, which results in a more representative sample of the microglial population being assessed. Automated tracing methods may struggle with densely packed microglia and fail to trace them correctly or not at all, thus losing an experimentally relevant population of microglia. However, adjustments and improvements to segmentation algorithms maybe be able to mitigate this issue in the future. Additionally automated tracing is more prone to “miss” fine terminal processes compared to manual/semi-automated methods due to the apparent breaks in terminal processes which occur because of thinning staining. In our experience of implementing automated tracing platforms, we have found it useful to include a filtering step for all morphological data that they output, to remove biologically unfeasible data points. Typically, we set a maximum expected value for each morphological parameter and remove all data obtained from any microglia which exceeds the limit in any single morphometric category e.g., cell volume. Despite the automated tracing platforms discussed above being extensively validated and capable of producing extremely accurate traces in a high throughput manner, they all suffer to varying degrees with issues pertaining to user friendliness and accessibility for operators with limited background in coding. None of them can be run from simple executable file akin to ImageJ and have limited implementation of graphical user interfaces. In some cases, in the original publications there was no clear signposting of where script repositories can be found and required the potential users to manually seek out the GitHub account of the authors. If automated tracing platforms are to be widely used and become a viable alternative to software packages such as Imaris Microscopy Image Analysis and Neurolucida 360^®^, further concerted development of one or more of these existing platforms is paramount. Such development should focus on improving user friendliness, maintaining/updating the software’s features and improving its uptake within the wider research community, whether this be through commercialisation or promotion as open-source software by a large research body such as the NIH as was done with ImageJ.

### Analyzing microglial morphometrics

The simplest method for analyzing microglial traces is comparing raw morphometric parameters obtained from them, between experimental groups. Any changes in morphometrics observed between groups can be used to infer whether any changes in microglial classification/activation have occurred. Morphometrically there are many features that can be extracted from microglia. As mentioned throughout, some of the most common parameters are cell soma, cell volume, ramification reflected by Sholl analysis (proximal and distal processes to the cell), number of terminal points, the total length of all processes and the territorial coverage of the microglia. When these parameters are combined, their relevancy and usefulness provide a more detailed interpretation of the microglia in the relative context. For example, an increase in both soma volume and a decrease in the number of branch points would suggest a shift toward microglial activation and therefore increased phagocytosis in the given condition (Leyh et al., 2021). Furthermore, several researchers have highlighted the importance of subtle fluctuations in microglial morphology in both the healthy and pathological brain. For example, where relative soma volumes and proximal processes remain constant but changes are observed in distal processes, which infer changes to microglia surveying activity or their support of synaptic plasticity (Tremblay et al., 2010; Karperien et al., 2013; Hristovska and Pascual, 2016). Attempts have been made to combine several morphometrics into one parameter which can be used to quantify microglial activation; ramification index is one such example. Ramification index is commonly found in the literature, however, no consensus has been reached on how to calculate this metric, with researchers using an array of different definitions (Table 4), making interexperimental comparisons challenging. It is also important to note that comparisons of different methods for obtaining microglial morphometrics have revealed that there can be some differences in measured effect sizes depending on the method employed. This appears to be truest when performing solely fractal analysis or Sholl analysis (Green et al., 2022b). When comparing methods which reconstruct microglia in 3D, the parameters extracted from the same cell by different software packages are comparable for most morphometrics (number of endpoints, number of branch points and cell volume), with branch length measurements being the exception, exhibiting high variability across the three methods assessed (York et al., 2018). In this section we provide an overview of the methods for analyzing microglial morphometrics which go beyond considering the raw values in insolation and instead attempt to use them to classify/categorize microglia into morphotypes.

**TABLE 4:** The varied definitions of “ramification index” found within the literature.

Definition	References
$\frac{C\,e\,l\,l\,p\,e\,r\,i\,m\,e\,t\,e\,r}{C\,e\,l\,l\,a\,r\,e\,a}$	Madry et al., 2018b
$\frac{M\,a\,x\,i\,m\,u\,m\,n\,u\,m\,b\,e\,r\,o\,f\,S\,h\,o\,l\,l\,i\,n\,t\,e\,r\,s\,e\,c\,t\,i\,o\,n\,s}{\#\,P\,r\,i\,m\,a\,r\,y\,p\,r\,o\,c\,e\,s\,s\,e\,s}$	Morrison and Filosa, 2013; Simon et al., 2020; Clarke et al., 2021
$\frac{\#\,E\,n\,d\,b\,r\,a\,n\,c\,h\,e\,s}{\#\,P\,r\,i\,m\,a\,r\,y\,b\,r\,a\,n\,c\,h\,e\,s}$	Sołtys et al., 2001; Heindl et al., 2018
$\frac{C\,e\,l\,l\,a\,r\,e\,a}{C\,o\,n\,v\,e\,x\,a\,r\,e\,a}$	Schilling et al., 2001; Wittekindt et al., 2022
$\frac{4\,\pi \times C\,e\,l\,l\,a\,r\,e\,a}{C\,e\,l\,l\,p\,e\,r\,i\,m\,e\,t\,e\,r^{2}}$	Schilling et al., 2004; Wodicka et al., 2015
$\frac{C\,e\,l\,l\,p\,e\,r\,i\,m\,e\,t\,e\,r/C\,e\,l\,l\,a\,r\,e\,a}{2\times {\left(\pi /C\,e\,l\,l\,a\,r\,e\,a\right)}^{1/2}}$	Madry et al., 2018a; Kyrargyri et al., 2020
$\frac{C\,e\,l\,l\,t\,e\,r\,r\,i\,t\,o\,r\,y}{C\,e\,l\,l\,v\,o\,l\,u\,m\,e}$	York et al., 2018; Steffens et al., 2023

### Cluster analysis

Now that automated tracing platforms have facilitated the generation of large microglial morphometric datasets, new methods for analysis have emerged to take advantage of them. Clustering analysis or clustering, which is commonly used in in transcriptomics, groups objects based upon common characteristics and similarity. Clustering therefore appears naturally suited for microglial morphological classification. Verdonk et al. (2016) successfully implemented cluster analysis based upon two morphological descriptors: complexity index (CI) and covered environment area (CEA) extrapolated from morphometrics obtained from 20,000 microglia per group using a custom Acapella script. CI is the ratio between the number of segments of a cell and the number of primary ramifications, where a segment is defined as a length of process between two nodes. CEA is the total 2D surface area occupied by the shape formed by linking all the extremities of the cell. Principal component analysis (PCA) identified no correlation between these two features making them suitable for k-means clustering, in which cells are assigned to one of four subpopulations: SP1 (CEA^low^/CI^low^), SP2 (CEA^low^/CI^high^), SP3 (CEA^high^/CI^low^), and SP4 (CEA^high^/CI^high^). In this particular case the authors make no inferences on how these sub-populations relate to microglial activity but instead present the ratio between SPs as a single metric to compare microglial populations. Verdonk et al. (2016) saw no changes in SP proportions in mice treated with and without LPS when looking at microglia taken from across the brain. However, when microglial populations from different brain regions were examined separately differences in SP proportions were observed not only with LPS treatment but also between brain regions in control animals. For example, in the striatum, post-LPS, the proportion of cells in SP4 increased from 3 to 46%. MIC-MAC developed by Salamanca et al. (2019) also implements k-means clustering to stratify microglia based upon their morphologies but combines it with semi-automated acquisition of morphometrics. MATLAB based MIC-MAC generates two masks per cell, one which utilizes a machine learning based, heavy smoothing algorithm (ilastik, Berg et al., 2019)^8^ to segment and one from a detailed rendering which captures the fine detail. The two masks are combined to produce a 3D reconstruction from which 62 morphometrics are extracted. Following PCA driven dimensionality reduction, 21 parameters are used in k-means cluster analysis during which microglia were assigned to one of 10 clusters, the number of clusters being determined by knee-plot analysis. Following cluster analysis, a graphical user interface (GUI) within MIC-MAC can be used to inspect cluster homogeneity. MIC-MAC was developed using a dataset of 11,142 microglia obtained from four sources: the CA1 of 1-month and 12-month-old mice and from post-mortem hippocampi of Alzheimer’s disease (AD) patients and age-matched controls. All clusters contained microglia derived from both species, however, some clusters had a higher proportion of cells coming from one source than the other. When considering microglia from AD patients versus aged-matched controls, k-means clustering revealed an expected shift in morphological classification. Similarly, morphOMICs developed by Colombo et al. (2022) also uses hierarchical clustering analysis (HCA) to classify microglia morphologically without any *a priori* adherence to pre-existing microglial morphotypes. MorphOMICs’ developers use IMARIS to generate 3D reconstructions of microglia which does introduce some of the limitations and biases associated with semi-automated methods discussed earlier in this review, however, given that the software uses the commonly used.swc file format, morphOMICs could be combined with fully automated reconstruction methods which mitigate some of these issues. A topological morphology descriptor is used to generate persistence barcodes from these 3D reconstructions. Persistence barcodes retain as much information as possible about a cell’s morphology by summarizing the 3D-tree complexity, radial distance and branching patterns. The use of persistence barcodes has a distinct advantage over the use of single morphometric parameters which can be influenced by interdependency and only capture certain features of a tree. These advantages are evidenced by morphOMICs’ developers, who show that when using HCA, classical morphometrics such as process length and number of branching points, are unable to recapitulate the same morphotypic resolution achieved using persistence barcodes. These examples show that cluster analysis can be used to separate microglia into assemblies based upon their morphology, and in the case of Salamanca et al. (2019) combined it with a very powerful, high-throughput method to gather morphometrics. Both approaches presented above are relatively simple and reliable methods for comparing microglial morphology and are capable of quantifying known shifts in microglial activation post-injury/disease. However, they do not produce classifiers which fit with the classical descriptors used by the field at large (i.e., homeostatic, hyper-ramified, reactive, ameboid and rod) which may impede the method’s uptake and acceptance by the wider research community.

In contrast to this, Fernández-Arjona et al. (2017) developed a HCA where the resulting clusters are tied to the existing microglial morphological nomenclature. Using FracLac^9^ and 840 manually generated single cell image masks, the authors generated 15 morphometric parameters per cell from rats intracerebroventricularly injected with neuraminidase or saline. Microglial activation was confirmed through manual classification of microglia and an observed increase of IBA1/IL1β colocalisation following neuraminidase treatment. The Thorndike procedure and Calinski-Harabasz criterion were used to estimate the number of clusters required to best represent the data before linear discriminant analysis (LDA) was used as the first stage of the HCA. LDA identifies characteristics that have a discriminate function >90% and is capable of separating cells into different groups. Convex hull span ratio, cell circularity and convex hull area were identified by LDA as being important characteristics and were used to create a decision tree to separate microglia into four clusters. Subsequent PCA suggested that these clusters should be subdivided to create a total of 8 clusters based upon the parameters: convex hull and convex hull circularity. Following the development of the classification decision tree, the authors proposed that Cluster 1 represented a mixture of ramified and activated morphologies, Cluster 2 represented ramified morphology (“resting”) microglia, Cluster 3 represented an intermediate morphology (hyper-ramified) and Cluster 4 represented an activated morphology. The subdivision of these clusters via PCA complicated matters in this case as microglia began to segregate not based upon activation status but instead brain region. For example, Cluster 2.2 was localized exclusively to the hippocampus. Intriguingly, microglia in the hypothalamus of rats injected with saline were in the same cluster (1.1) as some microglia from the hippocampi of neuraminidase treated rats, which supports the idea of microglial heterogeneity in the brain and that the morphotype of “resting”/salient microglia may be region specific. The HCA performed by Fernández-Arjona et al. (2017) was an effective implementation of cluster analysis for microglial classification; however, they did not validate their model with any other datasets such as rats treated with LPS, to show that the classifier they have produced can be implemented in a range of biological scenarios. Furthermore, they do not consider how ameboid or rod-shaped microglia may be treated by their decision tree, probably because they are not present in the saline/neuraminidase dataset. However, these morphological groups are important to consider in injured and diseased brains such as post-TBI or during AD.

### Machine learning

The use of machine learning algorithms to classify cells based on their morphology is well established in oncology (Kourou et al., 2015). Researchers have now begun to implement similar methodologies to classify microglia based upon morphometrics generated by high-throughput automated image analysis. However, there is a clear difference in the requirements of a predictive model for assessing microglial activation and one used for cancer diagnostics. In machine learning classification two errors can occur: false positives and false negatives. Cancer diagnostics can less afford false negatives i.e., patients with cancer given the all-clear, than false positives i.e., healthy patient is sent for follow up tests which rule out cancer. To this end predictive algorithms used in oncology focus on achieving a high specificity (true negative rate) to the detriment of their sensitivity (true positive rate). However, in the case of models designed for microglial classification the positive prediction (e.g., a cell has a reactive morphology) has the same importance as the negative prediction (e.g., a cell does not have a reactive morphology), so both sensitivity and specificity are of equal importance and a balance must be struck between the two. Several machine learning algorithms have been recently developed to classify microglia based upon their morphology. One such algorithm was created by Leyh et al. (2021) and uses a convolutional neural network (CNN) to assign microglia to one of four pre-determined classes (ramified, activated, rod or ameboid). The CNN was developed using a microglial image dataset (obtained using a slide scanner) containing 4,000 cells, which was generated by manually selecting 1,000 of each morphological group from a set of manually selected images generated from leptin receptor deficient and wild type (WT) mice. Leptin receptor deficiency (*db/db* or *db/+*) is an established mouse model in which microglia have adopted predominantly a non-homeostatic morphology (reactive, rod or ameboid) (Dey et al., 2014; Arroba et al., 2016). The image dataset was split into three sections: training (70%), testing (15%), and validation (15%). The training dataset was used to train the CNN who’s within model optimisation was driven by the testing dataset, with the accuracy of the final model (95.56%) being estimated using the validation dataset. The model’s accuracy, when broken down for each morphology, reveals that it can identify ramified (97.22%) and ameboid (97.78%) with relative ease, whereas in comparison it struggles to separate rod (91.67%) and activated (95.56%) morphologies. The functionality of the final model was confirmed by assessing microglial activation in a mouse model of ischemia. The CNN was able to detect and quantify the predicted increase in the proportion of activated microglia known to occur in the hippocampus and neocortex 24 h post-ischemia. One of the CNN pipeline’s unique advantages is the lack of any requirement for 3D-reconstruction of microglia, instead preferring to use computer vision to extract the information required for classification. This streamlines the process and ensures a consistency of data that is input into the neural network, something which other platforms cannot offer.

Another novel implementation of machine learning to assess microglial morphological changes has been developed by Silburt and Aubert (2022). In their workflow, named MORPHological Identification of Outlier clUSters (MORPHEUS), a support vector machine (SVM) was trained to recognize the morphology of active microglia using images of hippocampal microglia obtained from mouse brains whose blood brain barrier had been disrupted through focused ultrasound (FUS) or from aged TgCRND8 mice (AD mouse model). MORPHEUS identifies clusters of active microglia, with clusters here referring to microglia which were close spatially, not in a statistical sense à la k-means or HCA. In order to do this, it requires four parameters: nu (hyperparameter for a multiclass support vector machine which reflects the number of normal observations which lie outside the classification decision boundary), gamma (hyperparameter for the radial-basis-function kernel), minimum cluster size and minimum neighbor distance. An optimal set of hyperparameters was determined as being the set which maximized the clustering of microglia from FUS and TgCRND8 mice and produced no clustering of microglia from controls. Further analysis of clusters observed in FUS and TgCRND8 mice divided them into “focal” and “proximal” microglia, where “focal” microglia are defined as being the cluster which is surrounded by less active “proximal” microglia. Analysis downstream of MORPHEUS enables researchers to compare the number and size of spatially distinct clusters between experimental groups which serves as a quantitative measure of microglial activation. Whilst MORPHEUS’ developers don’t explicitly state that it can extract the number of individual “focal,” “proximal” and “non-active” microglia which would enable more traditional comparison of microglial activation, it should be feasible to do so albeit with minor tweaks to the software. The approach taken by Silburt and Aubert (2022) is a novel approach to assessing microglial activation *in vivo*, and evaluating cells as a collectively entity rather than as individuals certainly has advantages, such as identifying the brain regions impacted in disease and post-injury which may have particular relevance in a clinical setting. However, in the case of some CNS infections, diseases and psychiatric disorders, microglial activation is more disparate and spread out across a wide area, which MORPHEUS would not be able to quantify in its current incarnation. If MORPRHEUS’s measures of spatial distribution of microglial activation could be combined with a more traditional approach which gathers raw morphometrics from individual cells, MORPHEUS could prove to be an even more powerful tool for quantitative assessment of changes in microglial morphology.

**FIGURE 5:**
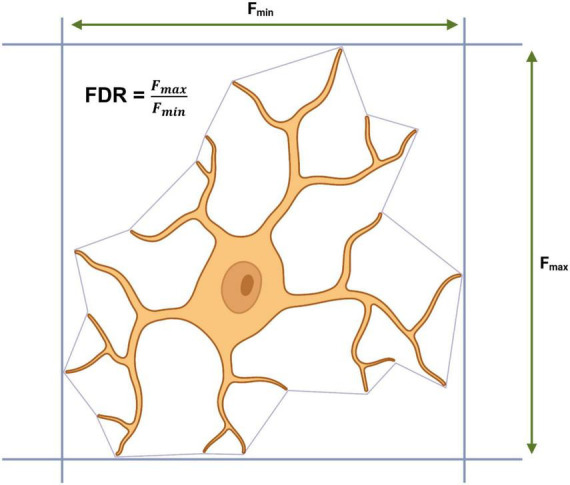
Calculating Feret’s maximum distance (F_max_), Feret’s minimum distance (F_min_) and Feret’s diameter ratio (FDR) from a convex hull.

Outside of the CNS, SVMs have also been used by Choi et al. (2022) to develop a supervised SVM classifier for retinal microglia, however, with a novel twist. The SVM was training exclusively using morphometrics extracted from microglia somas in the retinas of 2-, 6-, and 28-month-old mice. In order to do this, Choi et al. (2022) developed a pipeline containing a custom cell body counting script (ImageJ) to identify large masses of IBA1 staining and a custom auto-segmentation script (MATLAB) to separate out each soma into individual images. Using an exhaustive literature search, the authors generated qualitative descriptors of microglial somas for each the five classical morphotypes: ramified (small round circular), hyper-ramified (less circular than ramified, irregular, lobed and radially elongated), activated (larger soma than ramified, irregular and spatial restricted), rod-shaped (elongated narrow) and amoeboid (larger soma than ramified). From these qualitative descriptors, an initial set of quantitative predictors were generated for each morphotype based upon Feret’s maximum distance (F_max_ = greatest length between two tangents which are parallel on an object) and Feret’s diameter ratio (FDR = F_max_/F_min_), for example, rod microglia are defined as having an FDR > 3 and an F_max_ > 13.201 μm. Correct classification based upon F_max_ and FDR was confirmed by overlaying the soma over the original “complete” microglial image and additional parameters were extracted and used to define each morphotype (Figure 5). A final dataset containing 34 parameters from 1,200 somas (240 per morphotype) was used to train a linear SVM with a strong predictive power (true positive rate: >87.9%, false negative rate: >12.1%). The model identified an increase in the percentage of activated microglia in the retinas of aged mice (28-month-old), a finding that has previously been reported by other researchers using manual tracing in ImageJ (Damani et al., 2011). The authors do not confirm whether their cell body counting script and segmentation pipeline can extract morphometric data from microglial somas in the CNS. Microglia in the retina are arranged in a dual layer whereas microglia residing in the brain exist in a far more complex environment; this may pose a challenge to make Choi et al.’s (2022) approach suitable for use outside of the retina. However, the core principals of looking at microglial somas instead of the entire cell is intriguing and if it could be extended to microglia in the CNS, it would present a valuable addition to other automated methods for acquiring morphometrics and would increase the dimensionality of datasets available for cluster analysis and other classification algorithms.

### Community adoption

All the methods utilizing both cluster analysis and machine learning for microglial morphological classification have seen little to no uptake by the wider research community. Whilst it is difficult to know exactly why this has been the case, we would like to propose the following suggestions for ensuring wider use in future. Firstly, all algorithms developed for the classification of microglial morphotypes should be made available to all for use and for scrutinisation through GitHub repositories and a commitment from researchers to truly open research. Of the papers discussed: Salamanca et al. (2019), Colombo et al. (2022), and Silburt and Aubert (2022) provided a GitHub repository, or a dedicated website for their classifiers which were clearly signposted in their publications. In the case of other algorithms, GitHub links are not provided and instead the authors request interested parties contact them for access. However, in our experience, requests can produce mixed results, including in one case a flat refusal to share their code. Furthermore, a great drawback is that whilst the code may be made available, access is often not given to the datasets that were used to develop the model which renders third party validation and implementation of the algorithms in another experimental setting extremely difficult. Sharing of these datasets would also aid transparency and would give researchers a stable of cell morphology data that could be used in the training, testing and validation of their own prediction algorithms.

Secondly there is a significant barrier of entry for researchers who wish to implement machine learning into their experiments. All the methods presented above require an established familiarity with Python or MATLAB; the latter being especially problematic due to the requirement of a paid license and lack any GUIs or any other features to aid their use by novices. Whilst this perhaps has not been in the scope of previous projects, we propose that in the future consideration should be given to making published methods for classification simple to implement and with clear documentation to instruct other research groups such as an online tutorial. The benefits of machine learning driven microglial classification, such as reproducibility and inter-experimental comparisons, will only truly be felt when there is wide scale adoption and acceptance within the research community at large.

## Concluding remarks

Researchers have developed a variety of methods to quantitively analyze the morphology of microglia and thanks to rapid advances in automated acquisition of morphometrics, the field has changed drastically in the past decade. Despite significant strides in identifying new cellular markers (e.g., TMEM119 and HexB) and developing experimental models (e.g., CX3CR1^GFP^ and Sall1^GFP^) to visualize microglia *in vivo* and *ex vivo*, microglial morphological analysis needs to swiftly advance to capitalize on these methods to further the understanding of microglial biology. Manual classification will always have its place in the analysis of microglial morphology due to its simplicity and not requiring complex and/or expensive software packages. However, with time, manual classification may be resigned to the role of quality control for automated tracing methods and aiding the development of new classification algorithms. Likewise, analyzing microglial activation using raw morphometrics can be suitable for researchers studying diseases where microglia are activated *en masse* such as AD and TBI. However, for scenarios where low-grade activation of a small and perhaps distributed microglial subpopulation is expected, such as in models of major depressive disorder (Wang et al., 2022) and schizophrenia (Laskaris et al., 2016; Zhou et al., 2020), raw morphometrics alone will never be sufficient to detect subtle activity changes between experimental groups. To see the integration of classification algorithms into these kinds of research topics, developers need to implement features which lower the barrier of entry for their software to see the widespread uptake by the research community at large, a process which may require support from government research agencies such as the NIH and UKRI as was seen with ImageJ. The inter-experimenter reproducibility afforded by classification pipelines gives them a great advantage over manual classification and comparison of raw morphometrics. With these caveats the future looks bright for the integration of machine learning and cluster analysis into the microglia researcher’s biological toolkit. With this integration, analysis of more subtle changes in microglial activation across wide areas of the brain becomes possible, and the consistency of microglial morphological analysis is improved across the board.

## Author contributions

JR wrote the manuscript with support from PR, RB, JS, and MP. All authors contributed to the article and approved the submitted version.

## Footnotes

1
https://github.com/imagej/ImageJ

2
https://github.com/ivangtorre/multifrac

3
https://github.com/ElisaYork/3DMorph

4
https://github.com/Mahmoud-Abdolhoseini/GliaTrace-toolkits

5
https://github.com/isdneuroimaging/mmqt

6
https://github.com/Vaa3D/release/releases/

7
https://scikit-image.org/

8
https://www.ilastik.org/index.html

9
https://imagej.nih.gov/ij/plugins/fraclac/FLHelp/Introduction.htm#intro
